# Green Electrode Processing Enabled by Fluoro‐Free Multifunctional Binders for Lithium‐Ion Batteries

**DOI:** 10.1002/advs.202416995

**Published:** 2025-03-06

**Authors:** Xiuyu Jin, Ziting Zhu, Qiusu Miao, Chen Fang, Di Huang, Raynald Giovine, Linfeng Chen, Chaochao Dun, Jeffrey J. Urban, Yanbao Fu, Defu Li, Katie Liu, Yunfei Wang, Tianyu Zhu, Chenhui Zhu, Wei Tong, Gao Liu

**Affiliations:** ^1^ The Energy Storage and Distributed Resources Division (ESDR) Lawrence Berkeley National Laboratory Berkeley CA 94720 USA; ^2^ Pines Magnetic Resonance Center (PMRC)‐Core Facility College of Chemistry University of California Berkeley CA 94720 USA; ^3^ The Molecular Foundry Lawrence Berkeley National Laboratory Berkeley CA 94720 USA; ^4^ Advanced Light Source Lawrence Berkeley National Laboratory Berkeley CA 94720 USA; ^5^ School of Polymer Science and Engineering The University of Southern Mississippi Hattiesburg MS 39406 USA; ^6^ Department of Materials Science and Engineering Clemson University Clemson SC 29634 USA

**Keywords:** conjugated polymer, conductive binder, hierarchically ordered structure, lithium‐ion battery, green processing

## Abstract

The eco‐friendly processing of conjugated polymer binder for lithium‐ion batteries demands improved polymer solubility by introducing functional moieties, while this strategy will concurrently sacrifice polymer conductivity. Employing the polyfluorene‐based binder poly(2,7‐9,9 (di(oxy‐2,5,8‐trioxadecane))fluorene) (PFO), soluble in water‐ethanol mixtures, a novel approach is presented to solve this trade‐off, which features integration of aqueous solution processing with subsequent controlled thermal‐induced cleavage of solubilizing side chains, to produce hierarchically ordered structures (HOS). The thermal processing can enhance the intermolecular π–π stacking of polyfluorene backbone for better electrochemical performance. Notably, HOS‐PFO demonstrated a substantial 6–7 orders of magnitude enhancement in electronic conductivity, showcasing its potential as a functional binder for lithium‐ion batteries. As an illustration, HOS‐PFO protected SiOx anodes, utilizing in situ side chain decomposition of PFO surrounding SiOx particles after aqueous processing are fabricated. HOS‐PFO contributed to the stable cycling and high‐capacity retention of practical SiOx anodes (3.0 mAh cm^−2^), without the use of any conducting carbon additives or fluorinated electrolyte additives. It is proposed that this technique represents a universal approach for fabricating electrodes with conjugated polymer binders from aqueous solutions without compromising conductivity.

## Graphical abstract

Solubilizing side chains plays crucial roles in ensuring the aqueous processability of conjugated polymers. However, they do not need to remain intact postprocessing. Through controlled thermal‐induced cleavage of these side chains, hierarchically ordered structures can be generated, enhancing electrochemical performance and facilitating their utilization as conductive binders in lithium‐ion batteries.

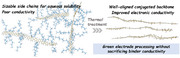

## 1 Introduction

**Figure 1:**
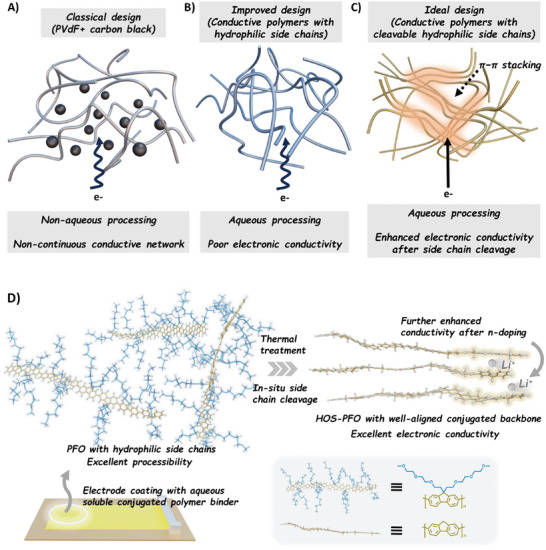
A) Illustration of the classical binder systems which consist of PVdF and conducting carbon particles. B) Illustration of aqueous‐processable conjugated polymer binder, which partially scarifices the electronic conductivity due to solubilizing side chains. C, D) Illustration of the molecular architecture and processing pathways of poly(2,7‐9,9 (di(oxy‐2,5,8‐trioxadecane))fluorene) (PFO) with thermal‐cleavable glycol side chains for green electrode processing.

The swift advancement of lithium‐ion batteries (LIBs) has established it as a leading and mature power source for a wide range of applications, from consumer electronics, and electric vehicles to grid‐scale energy storage. Over the past two decades, substantial efforts have been made to develop new electrodes (e.g., silicon as anodes) and electrolytes (solid‐state electrolytes) to further improve energy and power density.^[^ ^1^ ^]^ However, challenges for their large‐scale application persist in improving the cycling efficiency, capacity retention, and mechanical stability of these materials.^[^ ^2^ ^]^ In electrode processing, poly(vinylidene difluoride) (PVdF) is commonly used as a binder, and carbon materials such as acetylene black and Super P serve as conducting agents (**Figure** **1**A). The resulted unevenly distributed conductive phases and poor electronic conductivity are often the bottlenecks for electrode performance, especially for those anodes with significant volume change upon repeated charge and discharge.^[^ ^3^ ^]^

Alternatively, multifunctional conjugated polymer binders can simultaneously offer adhesion, elasticity, and electrical conductivity for battery electrodes, presenting a simple and cost‐effective solution to address interfacial reactions as well as conductivity issues caused by the volume expansion and contraction of active materials.^[^ ^4^ ^]^ To promote the applications of multifunctional conjugated polymer binders, it is important to adopt green manufacturing practices and mitigate the environmental impact associated with large‐scale electrode production.^[^ ^5^ ^]^ Unfortunately, unlike the typical hydrophilic binders (e.g., poly(acrylic acid) (PAA), cellulose),^[^ ^6^ ^]^ the aqueous solubility of common conductive polymers (e.g., polyacetylene,^[^ ^7^ ^]^ polyaniline (PANI),^[^ ^8^ ^]^ and polypyrrole^[^ ^9^ ^]^) are typically low due to a hydrophobic, spatially extended π‐bonding backbone. Although most conjugated polymers do not carry toxic polyfluoroalkyl structures, the common solvents employed for their processing are limited to halogenated and aromatic compounds (e.g., chlorobenzene, o‐dichlorobenzene), which are known to be harmful to both the environment and human health.^[^ ^10^ ^]^

To enhance the processability of conductive polymer binder in green solvents, especially aqueous solution (e.g., water, ethanol), the development of polymer blends is a well‐established strategy. Among electrical conductive polymers, poly (3,4‐ethylenedioxythiophene): poly (styrene sulfonate) (PEDOT: PSS) has become the gold standard with high stability and conductivity,^[^ ^11^ ^]^ and the charge‐balancing dopant PSS allows the composite dispersity in aqueous solution.^[^ ^12^ ^]^ However, polymer blends such as PEDOT: PSS could only be dispersed in aqueous solution as a form of nanoparticles instead of individual polymer chains, which results in significant colloidal stability issues during long‐term storage and transport.^[^ ^13^ ^]^ Furthermore, the agglomeration of nanoparticles and the incomplete binder coverage on active materials failed to fully address the interface instability. Consequently, the use of toxic and persistent fluorinated electrolyte additives, such as fluoroethylene carbonate (FEC), remains necessary to optimize the solid electrolyte interface (SEI).^[^ ^14^ ^]^

As another option, it is a natural perception to conduct chemical modifications and introduction of solubilizing polar side chains on conjugated polymers.^[^ ^9^, ^15^ ^]^ These approaches aim to disrupt intermolecular interactions and increase the flexibility of the polymer chains, ultimately improving their solubility and processability for practical applications. Numerous hydrophilic moieties with nonionic, anionic, cationic, or zwitterionic polyvalent structures have been demonstrated powerful in improving the water solubility of conjugated polymers.^[^ ^15^, ^16^ ^]^ However, the sizable side chains can create steric hindrance, limiting the close packing and intermolecular interactions between polymer chains (Figure 1B). This can disrupt the formation of ordered structures, reducing the degree of crystallinity, and, consequently, the electrical conductivity.^[^ ^17^ ^]^ The presence of bulky side chains may also introduce torsional disorder along the polymer backbone. This disruption in planarity can impede the delocalization of π‐electrons, which does not support efficient charge transport in conjugated polymer binders.^[^ ^18^ ^]^ Therefore, in most previous reports on aqueous processable conjugated polymer binders (Table S1, Supporting Information), the addition of conductive carbon additives was still necessary to achieve significant electrode conductivity.^[^ ^14^, ^19^ ^]^

To fulfill the solubility requirements without sacrificing functionality, we consider an innovative approach to synthesize conjugated polymer binder with cleavable solubilizing side chains, which could be selectively removed after aqueous electrode processing (Figure 1C). As a demonstration of this concept, we herein introduce an aqueous soluble conductive polymer, poly(2,7‐9,9 (di(oxy‐2,5,8‐trioxadecane))fluorene) (PFO), which consists of a polyfluorene backbone for electronic conductivity and hydrophilic oligo ethyleneoxide (EO) side chains for improving its solubility in protic solvents (Figure 1D). Without introducing a delicate reactive linker, the hydrophilic EO side chains themselves are thermal‐cleavable, which readily promotes the formation of a more densely packed polyfluorene backbone with hierarchically ordered structures (HOS). The HOS characteristics of the polymer binder significantly boost the electronic conductivity, circumventing the reliance on any conventional conducting carbon additives or fluorinated electrolyte additives.

## 2 Results and Discussion

**Figure 2:**
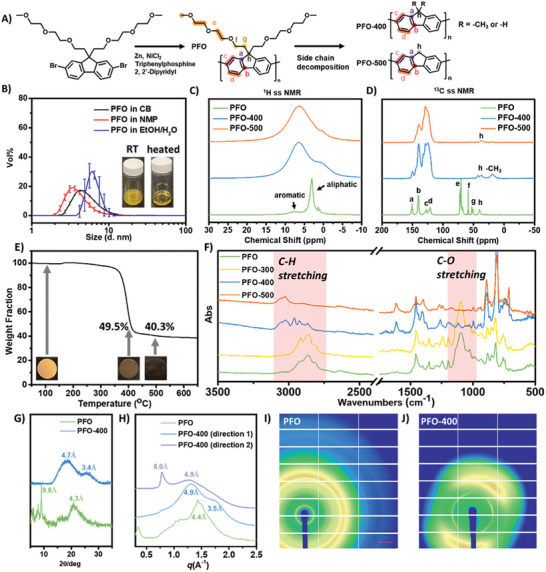
Solvent processing and thermal processing of PFO. A) Preparation of PFO polymer via straightforward Yamamoto coupling reaction of 2,7‐dibromo‐9,9(di(oxy‐2,5,8‐trioxadecane)) fluorene, as well as the illustration of controlled side chain decomposition of PFO at different temperature. B) The hydrodynamic size (D_h_) distribution of PFO in various solvents and conditions. Inset: the appearance of PFO in ethanol/water mixture at different temperatures. C) The solid‐state ^1^H NMR spectra of PFO, PFO‐400, and PFO‐500. D) The solid‐state ^13^C NMR spectra of PFO, PFO‐400, and PFO‐500. Assignments of the observed ^13^C site in PFO are indicated from a to h as well at the terminal CH_3_ group formed in PFO‐400. E) The pyrolysis behavior of PFO was characterized by the TGA. Temp ramp rate = 2.5 °C min^−1^, under Argon atmosphere. F) The evolution of ATR‐FTIR spectra of PFO during the pyrolysis process. G) The XRD patterns of PFO films and 400 °C processed PFO films, samples were prepared from the ethanol‐water mixture. H) The WAXS 1D patterns of PFO films and 400 °C processed PFO films, samples were prepared from the ethanol‐water mixture. I, J) The 2D WAXS pattern of PFO and 400 °C processed PFO films.

The synthesis of poly(2,7‐9,9 (di(oxy‐2,5,8‐trioxadecane)) fluorene) (PFO) was carried out using a straightforward Yamamoto coupling reaction of 2,7‐dibromo‐9,9(di(oxy‐2,5,8‐trioxadecane)) fluorene (**Figure** **2**A), as detailed in prior literature.^[^ ^20^ ^]^ Notably, this synthesis did not involve the use of an expensive palladium catalyst, opening up more possibilities for low‐cost and scaled‐up production. Confirmation of the successful synthesis was achieved through proton nuclear magnetic resonance (^1^H‐NMR) analysis (Figure S1A, Supporting Information). Gel permeation chromatography (GPC) analysis revealed a monomodal molecular weight distribution of PFO (M_n_ = 11.0 kDa, PDI = 2.6, Figure S1B, Supporting Information).

The resulting polymer exhibited excellent solubility in both aprotic and protic solvents. In chlorobenzene (CB) and N‐methyl pyrrolidone (NMP), PFO formed a homogeneous solution, as verified by dynamic light scattering (DLS) results (Figure 2B). The hydrodynamic size (D_h_) was ≈4.3 nm and 3.4 nm, respectively, indicating an absence of aggregation. In aqueous solutions like ethanol/water (v:v = 2:1) mixtures, PFO (5 wt.%) initially formed a cloudy dispersion at room temperature, despite its size could not be accurately quantified. However, at elevated temperatures (>35 °C), PFO transitioned to a clear solution. The D_h_ of PFO in ethanol/water was ≈13.5 nm at 35 °C, indicating minimal aggregation of PFO molecules. A decrease in D_h_ to 7.5 nm occurred at 45 °C, likely attributed to the disruption of intermolecular hydrogen bonding. Further temperature increase (e.g., 50 °C) resulted in a monomodal size distribution ≈6.0 nm for the PFO sample in ethanol/water, indicating individual dispersity (Figure S2, Supporting Information). Therefore, for processing the PFO in aqueous solution, appropriate pre‐heating sample is necessary to ensure a uniform film formation.

The EO side chain structures not only introduced compatibility of the polymer to aqueous solution but also resulted in excellent materials crystallinity which potentially benefits the electrochemical properties. We used polarized light microscopy (PLM) to characterize the evolution of the appearance of PFO droplet (in ethanol/water 2:1 mixture) upon air‐drying at room temperature. Birefringence was observed within 10 min (Figure S3, Supporting Information), indicating the anisotropic nature of the formed structures. Additionally, X‐ray diffraction (XRD) patterns of PFO (Figure 2G; Figure S4, Supporting Information) identified peaks at 2θ = 9.05°, corresponding to a d‐spacing of 9.8 Å, in line with the contour length of the glycol groups oriented perpendicular to the polymer's main chain. These findings suggest a lamellar patterned d‐spacing for PFO. The strong, broad diffraction peaks at ≈2θ = 20.5° for PFO correspond to π–π stacking distances of 4.3 Å, resembling distances observed in other fluorene‐based polymeric and oligomeric semiconductors.^[^ ^21^ ^]^ The crystallinity of PFO was also confirmed by the wide‐angle X‐ray scattering (WAXS) results (Figure 2H; Figure S5, Supporting Information), as a sharp peak at 4.4 Å was observed, which echoes well with the XRD results.

After the solvent processing, we proceeded to examine the thermal‐induced side‐chain cleavage characteristic of PFO, given that the electronic conductivity of polyfluorene derivatives could be significantly improved by selectively removing solubilizing moieties and generating hierarchically ordered structures (HOS).^[^ ^22^ ^]^ It was confirmed that the EO side chains of PFO could be readily removed via facile thermal processing, which was evident in the thermogravimetric analysis (TGA) of PFO conducted under an Argon atmosphere (Figure 2E). The thermal decomposition was initiated at 350 °C, with the majority of PFO's side chains effectively removed at 400 °C, resulting in a 49.5% weight retention. For a better understanding of the PFO structural evolution, a solid‐state NMR spectroscopy study was performed, and the proposed structures of PFO‐400 (PFO processed at 400 °C) and PFO‐500 (PFO processed at 500 °C) are described in Figure 2A. Upon increasing the thermal processing temperature, the net decrease fraction of aliphatic ^1^H signals confirmed the loss of side chains (Figure 2C). Meanwhile, using the Cross‐Polarization Magic Angle Spinning (CPMAS) technique, the ^13^C NMR provided well‐resolved spectra for composition and structure identification (Figure 2D). In PFO, the ^13^C signals from the aliphatic side chains are observed between 35 to 75 ppm while the PFO backbones ^13^C signals are observed between 115 and 155 ppm (see Figure 2A,D for the exact ^13^C signal assignment). After thermal treatment, ^13^C signals from the side chains are reduced. It is also worth noting that after thermal treatment the PFO backbone ^13^C signature exhibits a much wider line shape that could suggest a more densely packed structure.

The *C‐9H* signal (peak h) of PFO‐400 exhibited bimodal distribution from 42.7 to 36.9 ppm, demonstrating the decomposition of side chains and generation of new C─H bonds at *C‐9* position. Additionally, a new peak at 19.0 ppm was observed in PFO‐400, corresponding to the formation of ─CH_3_ group after side chain decomposition. In comparison to PFO‐400, the PFO‐500 exhibited a monomodal *C‐9* peak at 36.9 ppm, suggesting complete removal of side chains.

Attenuated total reflectance‐Fourier transform infrared spectroscopy (ATR‐FTIR) results also support the structural elucidation. No significant spectral changes were observed when the temperature was raised to 300 °C (Figure 2F). In contrast, PFO‐400 exhibited a substantial reduction in C─H stretching signals within the range of 2800–2900 cm^−1^ as well as the C─O stretching signals at 1100 cm^−1^, indicative of the elimination of side chains containing ethylene oxide units. Additionally, a new peak at ≈3040 cm^−1^ appeared, signaling the formation of C9‐*H* sites within the fluorene ring. Elevating the temperature from 400 to 500 °C led to the complete loss of PFO functionalities, as no C‐H signals within the range of 2800–2900 cm^−1^ remained. This process is accompanied by a color change from brown to black, suggesting partial carbonization of the materials.

The removal of EO side chains promoted the formation of HOS structures, which was confirmed by the XRD results. PFO‐400 sample demonstrated a shoulder peak ≈2θ = 26.0° (Figure 2G; Figure S4, Supporting Information), and the corresponding d‐spacing was close to the ideal π–π stacking distance (3.4 Å, twice the estimated van der Waals (vdW) radius of carbon (1.7 Å)). Similarly, the WAXS characterization also validated the well‐packed polymer chains. A shoulder peak at 3.5 Å evolved (Figure 2H; Figure S5, Supporting Information), echoes well with the XRD results. Moreover, unlike the pristine PFO, the HOS‐PFO demonstrated significant anisotropy (Figure 2I,J), further suggesting the well‐alignment of polymers after the thermal processing at 400 °C.

**Figure 3:**
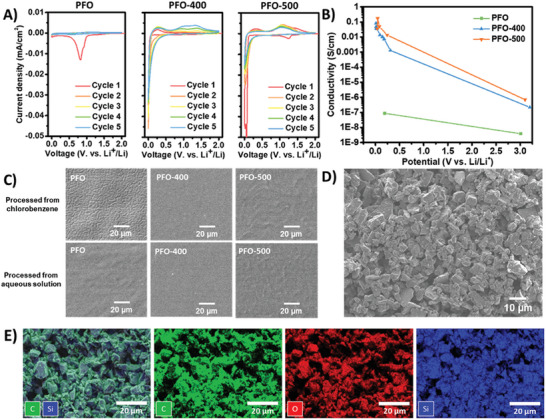
A) Cyclic voltammetry study of pristine PFO, PFO‐400 and PFO‐500. Polymers were processed from an ethanol/water 2:1 mixture. The scan rate is 0.01 mV s^−1^ to ensure complete lithiation of the polymers. B) The relationship between HOS‐PFO conductivity and its potential after lithiation. C) The surface morphology of PFO and HOS‐PFO processed from different solvents. D,E) SEM image and energy dispersive X‐Ray spectroscopy (EDS)‐based elemental mapping results of SiOx‐PFO‐aq‐400 (theoretical loading ≈1.0 mg SiOx/cm^2^) fabricated from ethanol/water (v:v = 2:1).

The thermal processing temperature deeply influenced the electrochemical characteristics of produced HOS‐PFO. Initially, cyclic voltammetry (CV) was employed to assess the electrochemical behavior of pristine PFO, PFO‐400, and PFO‐500. Pristine PFO exhibited a notable irreversible peak at 0.83 V, likely attributed to glycol side chain reduction rather than lithiation (**Figure** **3**A). Decreasing the potential to 0 V did not yield any current response, indicating the poor *n*‐doping ability and conductivity of pristine PFO. Conversely, PFO‐400, with partially retained side chain functionality leading to enhanced π–π orbital overlapping, demonstrated significantly improved lithiation capabilities compared to pristine PFO (Figure 3A). Furthermore, a gradual increase in lithiation capacity was observed over repeated cycles, suggesting sluggish initial lithiation kinetics. This phenomenon was corroborated by potential hold experiments, showing an initial rise followed by a decline in the current (Figure S6, Supporting Information). In contrast, PFO‐500 exhibited much faster lithiation kinetics, evident from the rapid current decay during potential hold at 10 mV versus Li^+^/Li, likely attributed to completely removed side chains facilitating unimpeded Li^+^ movement along the polymer layers. Although the initial lithiation capacity surpassed that of PFO‐400, a significant fade was noted in PFO‐500 after repeated cycling (Figure 3A), underscoring the necessity to partially retain functionalities. This preservation ensures sufficient molecular flexibility and material cyclability for practical electrochemical applications.

To quantify the electronic conductivity of HOS‐PFO, the materials were also prepared on interdigital electrodes via sequential coating and thermal processing. It was found pristine PFO without thermal treatment has limited conductivity without doping, and it could not be improved via lithiation; In contrast, the PFO‐400 showed a 6 magnitude conductivity increase from 2.2 × 10^−7^ to 0.081 S cm^−1^ after complete lithiation (Figure 3B), suggesting its good capability for interface charge transfer after cleaving the side chains. With further elevated thermal processing temperature, PFO‐500 demonstrated a faster doping process. However, deep lithiation did not result in significantly higher conductivity, as after bringing potential from 45 to 38 mV, its apparent conductivity decreased from 0.17 to 0.037 S cm^−1^, which echoes well with the cyclic voltammetry results, suggesting possible cracking or delamination due to poor flexibility of polymer chains.

Because thermal‐induced side chain cleavage may impact their ionic conductivity as Li^+^ transport through vehicular motion is more difficult, we quantified the ionic conductivity of HOS‐PFO polymers produced from different thermal treatments (Figure S8, Supporting Information). Based on the EIS study, it was found both PFO‐400 and PFO‐500 did not exhibit noticeable ionic conductivity without lithiation, however, their ionic conductivity was improved to 10^−7^–10^−6^ S cm^−1^ after significant lithiation (<0.2 V vs Li/Li^+^). We hypothesize that in the case of HOS‐PFO polymers, the lithium‐ion diffusion was not controlled by the segmental movement of polymer chains. Instead, the lithium ions migrate in between the stacked polyfluorene backbones, which is similar to the case of graphite. As HOS‐PFO polymers became more lithiated, their ionic conductivity generally increased, reaching a peak at a moderate level of lithiation before decreasing slightly as the polymers reached full lithiation; This is possibly because the intercalated lithium ions facilitate the movement of additional lithium ions within the polymer structure, but excessive lithiation can lead to lattice strain and hinder ion mobility.

In addition to affecting the polymer's electrochemical characteristics, the thermal processing temperature also determined the polymer's surface morphology. Nonuniformity is a limiting factor for the performance of devices which often leads to variations in charge transport properties and results in reduced device performance.^[^ ^23^ ^]^ Like many other conjugated polymers, PFO also demonstrated a tendency to aggregate or form periodic wrinkles during the drying process, however, the controlled thermal processing demonstrated the potential to release the residual stress and promote the formation of continuous, uniform HOS‐PFO structures. We cast PFO films from various solvents (CB, NMP, and ethanol/water 2:1 mixture) and allowed them to dry on a silicon wafer at room temperature. Scanning electron microscopy (SEM) analysis revealed distinct surface patterns and wrinkles for each solvent‐cast PFO film (Figure 3C; Figure S9A, Supporting Information), likely attributable to different solvent polarity and evaporation rates. However, thermal processing at 400 °C eliminated these features, yielding smooth PFO‐400 samples without cracking (Figure 3C; Figure S9B, Supporting Information). This phenomenon is likely due to significant weight loss (>50%) during side chain decomposition and polymer reorganization. It was worth mentioning that the thermal processing conditions must be chosen carefully to obtain a smooth HOS‐PFO film, as elevating the heating temperature to 500 °C induces surface wrinkling in PFO‐500 films, possibly due to partial carbonization.

With the high conductivity, we further validated the application of different HOS‐PFO as polymer binder materials in lithium‐ion batteries. We used PFO as the binder to fabricate SiOx‐PFO anodes from slurry with ethanol/water (v:v = 2:1) as the solvent, and they were processed at 400 °C (SiOx‐PFO‐aq‐400) and 500 °C (SiOx‐PFO‐aq‐500) to produce in situ formed HOS‐PFO structures surrounding SiOx active materials. Noticing that the charge carrier transport fully relied on the HOS‐PFO, as no conductive carbon additives were added to the electrode composite. Both SiOx‐PFO‐aq‐400 and SiOx‐PFO‐aq‐500 exhibited uniform morphology (Figure 3D; Figure S10, Supporting Information), and the HOS‐PFO was homogeneously dispersed in the electrode (Figure 3E; Figure S10, Supporting Information). SiOx‐PFO‐aq‐500 exhibited higher adhesion compared to SiOx‐PFO‐aq‐400 during the 180° peeling test (Figure S11, Supporting Information), possibly due to the surface reaction between the copper current collector and the polymers at elevated temperatures. However, the SiOx‐PFO‐aq‐400 is still the preferred selection for electrode fabrication, as PFO‐400 demonstrated better elasticity (Figure S12, Supporting Information), which is essential for maintaining the material's integrity considering the volume expansion of SiOx during cycling.

**Figure 4:**
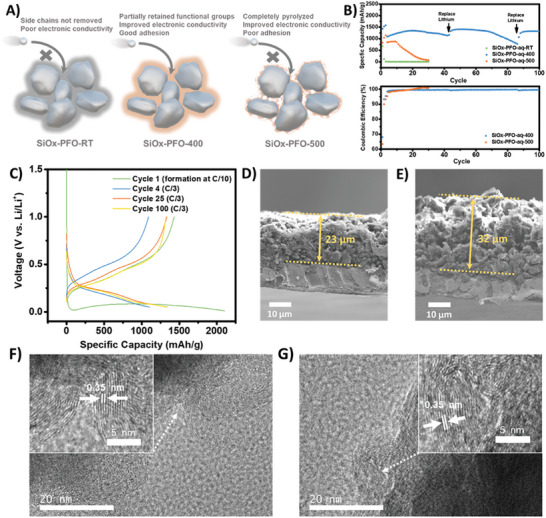
Half‐cell performance of SiOx‐PFO anodes (theoretical loading ≈1.0 mg SiOx/cm^2^). A) Illustration of the relationship between side chain availability of PFO/HOS‐PFO and cyclability of SiOx electrodes. B) The capacity and Coulombic efficiency evolution of the Li||SiOx‐PFO half cells. It is worth mentioning that we selected a baseline electrolyte without FEC additive for cell evaluation, and this is likely the primary reason why replacing the Li metal is necessary during our half‐cell tests.^[^ ^24^ ^]^ C) Capacity‐voltage profile for Li||SiOx‐PFO‐aq‐400 half cell. Initial Coulombic efficiency was 68.1%. D) The cross‐section SEM image of SiOx‐PFO‐aq‐400 anode before cycling. E) The cross‐section SEM image of SiOx‐PFO‐aq‐400 anode after being cycled 100 times. F) High‐resolution TEM image of SiOx‐PFO‐aq‐400 before cycling. G) High‐resolution TEM image of SiOx‐PFO‐aq‐400 after being cycled 100 times.

It was found the thermal processing temperature could significantly influence the electrode performance. The capacity of the electrode without heating (SiOx‐PFO‐aq‐RT) decayed very quickly due to the deficient electronic and ionic transport (**Figure** **4**B). Fast capacity fade of the SiOx‐PFO‐aq‐500 was also observed after ten cycles, resulting from the insufficient flexibility and weak adhesion of the polyfluorene backbone to the SiOx surface, as illustrated in Figure 4A. In comparison, the SiOx‐PFO‐aq‐400 demonstrated stable performance, with unchanged cyclability after 100 cycles (Figure 4B,C), due to partially retained side‐chain functionality and polymer flexibility.

After cycling, the swelling ratio of the electrode was 139% (Figure 4D,E), which is comparable to the literature‐reported values.^[^ ^25^ ^]^ Meanwhile, it was found the solvent selection for SiOx‐PFO slurry preparation did not bring much difference, as SiOx‐PFO‐cb‐400 electrode prepared from CB also demonstrated uniform morphology (Figure S15, Supporting Information) and similar electrochemical performance (Figure S16, Supporting Information).

We also confirmed the unchanged crystal structure of the PFO‐400 after long‐term cycling, which enabled the excellent cyclability of the SiOx‐PFO‐aq‐400 electrode. The high‐resolution transmission electron microscopy (HR‐TEM) clearly resolved the wavy lattice fringe of PFO‐400 (Figure 4F,G). The unchanged polymer d‐spacing was observed, demonstrating that the conductive binder still maintained its structure after cycling.

Controlled experiments were also performed to compare HOS‐PFO with classical binders. Non‐conductive binders including PAA and PVDF were selected, and conductive additives including carbon black and graphite were introduced. Both samples demonstrated fast capacity fading after 20 cycles (Figure S17, Supporting Information). Another controlled sample (SiOx‐PAA‐PANI) with PAA and polyaniline as binders also exhibited insufficient cyclability, possibly due to poor solubility and processibility of PANI in the slurry. The comparative analysis results demonstrated the superior performance of PFO as the binder for SiOx anode materials.

**Figure 5:**
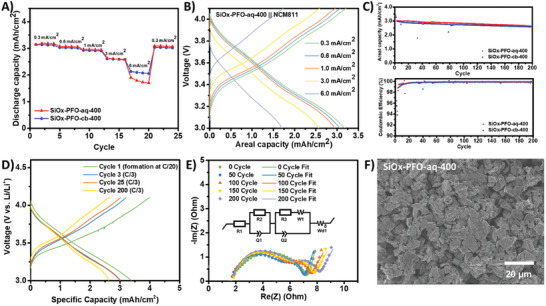
Full cell performance of practical SiOx‐PFO anodes (theoretical loading ≈3.0 mg SiOx/cm^2^). A) Rate capability for high‐loading practical SiOx‐PFO‐400||NCM811 full cells. Charge rate and discharge rate were controlled at the same. B) Charge‐discharge profile of SiOx‐PFO‐aq‐400||NCM811 full cell at different current densities. C) Capacity and Coulombic efficiency evolution of SiOx‐PFO‐aq‐400 and SiOx‐PFO‐cb‐400 during full cell test. D) Capacity‐voltage profile for SiOx‐PFO‐aq‐400||NCM811 full cell. Initial Coulombic efficiency was 85.0%. E) The electrochemical impedance spectroscopy results of SiOx‐PFO‐aq‐400||NCM811 full cell at different stages. F) The surface morphology of SiOx‐PFO‐aq‐400 samples after full cell cycling for 200 cycles.

Uncovered the optimized performance of PFO‐400 as the binder for silicon anode, furthermore, SiOx‐PFO‐cb‐400 and SiOx‐PFO‐aq‐400 with 86 wt.% of m‐SiOx were fabricated for practical (3.0 mAh cm^−2^) full‐cell cycling with high‐nickel content cathode, NMC811, to evaluate the potential for practical cell manufacturing. Both full cells support high‐rate cycling. The SiOx‐PFO‐aq‐400 demonstrated slightly lower, but comparable rate performance compared to SiOx‐PFO‐cb‐400 (**Figure** **5**A,B), and over 80% of charge capacity was delivered for cycling at 1 C (3.0 mA cm^−2^), with a Coulombic efficiency of 99.3%. Figure 5C,D shows the capacity retention of full cells cycling at a rate of 0.33 C, after electrochemical pre‐lithiation of the anode materials. It was found that the SiOx‐PFO‐aq‐400 processed from ethanol/water still retained 86% capacity after 200 cycles with an average Coulombic efficiency of 99.83%, which is comparable to the SiOx‐PFO‐cb‐400 processed from CB. Electrochemical impedance spectroscopy (EIS) was also used for monitoring the interface evolution during the long‐term cycling, and a very small semicircle in the mid‐frequency region was observed, indicating good contact between SiOx anode materials and conductive PFO‐400 binder (Figure 5E). Meanwhile, a minimum impedance increase was observed over 200 cycles, suggesting that the PFO‐400 binder may serve as a uniform and stable protection layer on the SiOx anode materials upon repeated lithiation and de‐lithiation process. Both SiOx‐PFO‐aq‐400 and SiOx‐PFO‐cb‐400 showed retained morphology without cracking after cycling, indicating good mechanical strength of the PFO‐400 (Figure 5F; Figure S18, Supporting Information).

## 3 Conclusion

Utilizing polyfluorene‐based PFO as a model, we have introduced an innovative strategy to achieve green electrode processing with multifunctional conjugated polymer binders. PFO, featuring solubilizing EO side chains, exhibited exceptional solubility across a variety of aprotic solvents and aqueous solutions. Following processing from an ethanol/water mixture, the selective removal of EO side chains via pyrolysis enhanced the intermolecular π–π stacking of the resulting HOS‐PFO, thereby concurrently improving its electrochemical performance. Among the various HOS‐PFO samples, PFO‐400 with partially retained functionality demonstrated a balanced combination of material flexibility, surface affinity, and electronic conductivity. Notably, it showed significant enhancement in electronic conductivity upon *n*‐doping, indicating excellent compatibility with lithium‐ion battery anodes such as SiO_x_. Thermal processing of SiO_x_‐PFO electrodes can in situ generate highly conductive HOS‐PFO surrounding the SiO_x_ particles, aiding in maintaining electrical contacts and material integrity under significant volume changes during battery operation. As evidenced by our results, practical (3.0 mAh cm^−2^) SiOx‐HOS‐PFO anodes exhibited ≈86% capacity retention after 200 cycles at 0.33 C. To the best of our knowledge, our work is the only example that fulfills all of these criteria: no conductive carbon additives in the electrode, no electrolyte additives, micro‐sized active material, and low binder usage. We posit that this methodology offers new opportunities for the design and green processing of conductive polymer binders for batteries while preserving their elasticity and conductivity.

## Conflict of Interest

The authors declare no conflict of interest.

## Supporting information



Supporting Information

## Data Availability

The data that support the findings of this study are available in the supplementary material of this article.
