# Supplementary material for: Green Electrode Processing Enabled by Fluoro‐Free Multifunctional Binders for Lithium‐Ion Batteries
Source: Adv Sci (Weinh). 2025 Mar 6;12(17):2416995. doi: 10.1002/advs.202416995 (PMC12061235; doi:10.1002/advs.202416995)
Supplement: Supplementary file 1 — Supporting Information [file ADVS-12-2416995-s001.docx]

**Supporting information**

**Green Electrode Processing Enabled by Fluoro-Free Multifunctional Binders for Lithium-ion Batteries**

Xiuyu Jin,^[a]^ Ziting Zhu,^[a]^ Qiusu Miao,^[a]^ Chen Fang,^[a]^ Di Huang,^[a]^ Raynald Giovine,^[b]^ Linfeng Chen,^[c]^ Chaochao Dun,^[c]^ Jeffrey J. Urban,^[c]^ Yanbao Fu,^[a]^ Defu Li,^[a]^ Katie Liu,^[a]^ Yunfei Wang,^[d,e]^ Tianyu Zhu,^[f]^ Chenhui Zhu,^[d]^ Wei Tong,^[a]^ and Gao Liu*^[a]^

[a] X. Jin, Z. Zhu, Q. Miao, C. Fang, D. Huang, Y. Fu, D. Li, K. Liu, W. Tong, G. Liu.
The Energy Storage and Distributed Resources Division (ESDR)
Lawrence Berkeley National Laboratory, Berkeley, California 94720, United States
E-mail: gliu@lbl.gov

[b] R. Giovine

Pines Magnetic Resonance Center (PMRC) – Core Facility

College of Chemistry, University of California, Berkeley, California 94720, United States

[c] L. Chen, C. Dun, J. J. Urban.
The Molecular Foundry
Lawrence Berkeley National Laboratory, Berkeley, California 94720, United States

[d] Y. Wang, C. Zhu.
Advanced Light Source
Lawrence Berkeley National Laboratory, Berkeley, California 94720, United States

[e] Y. Wang.
School of Polymer Science and Engineering
The University of Southern Mississippi, Hattiesburg, Mississippi 39406, United States

[f] T. Zhu.
Department of Materials Science and Engineering
Clemson University. Clemson, South Carolina, 29634, United States

* Corresponding author. gliu@lbl.gov

**Materials and methods**

**Materials**

All chemicals for polymer synthesis were purchased and used without further purification. All materials utilized for the fabrication of the battery were procured from commercial sources. These include micro-sized SiOx particles (m-SiOx) obtained from Shin-Etsu Chemical Co., a lithium chip from MTI Co., Celgard 2400 separator from Celgard Co., a lithium-ion electrolyte consisting of 1.2 M LiPF_6_ in ethylene carbonate-ethyl methyl carbonate (EC:EMC) with a ratio of 3/7 w/w, specifically known as Gen 2 electrolyte and sourced from Argonne National Laboratory, and a lithium nickel manganese cobalt oxide (NCM811) cathode with a capacity of 4.0 mAh/cm^2^, produced by the CAMP Facility at Argonne National Laboratory.

**Spectroscopic Characterization.** Regular scanning electron microscopy (SEM) imaging was performed with JSM-7500F field emission scanning electron microscope with acceleration voltage at 15 kV. Energy dispersive X-Ray spectroscopy (EDS) and elemental mapping was performed with Zeiss Gemini Ultra-55 Analytical Field Emission Scanning Electron Microscope with acceleration voltage at 10 kV. Transmission Electron Microscopy (TEM) was performed with JEOL 2100-F Field-Emission Analytical Transmission Electron Microscope. Proton nuclear magnetic resonance (^1^H NMR) spectra of polymer was obtained on a Bruker Biospin Avance II 500 MHz NMR spectrometer using CDCl_3_ as deuterated solvent. Solid state NMR (ssNMR) spectra were obtained at *B*_0_= 9.4 T (400.1 MHz for ^1^H) using a from Bruker Biospin spectrometer equipped with an Avance IV NEO console and a 3.2 mm double resonance HX Magic Angle Spinning (MAS) probe spinning at 20 kHz. ^1^H and ^13^C chemical shift were referenced with respect to tetramethylsilane using the CH resonance of adamantane as a secondary external reference at δ_iso_(^13^C)= 38.48 ppm and δ_iso_(^1^H)= 1.8 ppm. ^1^H ssNMR spectra were obtained using a rotor synchronized spin-echo sequence (90° − τ_R_ − 180° − τ_R_ – AQ) with a 90° radio frequency (RF) pulse of 2.1 µs, a repetition time of 5 s and 16 transients. ^13^C ssNMR spectra were obtained using a ^1^H→^13^C Cross-Polarization (CP) transfer under MAS (CP-MAS). The CP-MAS experiments used a contact time of τ_CP_ = 4 ms, during which a constant RF-field equal to 75.4 kHz was applied on the ^13^C, while the ^1^H RF‑field amplitude was linearly ramped from 89.7 to 99.7 kHz. During ^13^C acquisition, high-power ^1^H decoupling was applied using the SPINAL-64 (Small Phase Incremental Alternation with 64 steps) ^1^decoupling scheme with an RF-field amplitude set to 89.5 kHz. A total of 1024 transients were averaged with a repetition time of 2 s resulting in experimental times of 35 min. All solid-state NMR data were processed using Bruker TopSpin 4.3.0. The hydrodynamic sizes of the samples were characterized via dynamic light scattering (Zetasizer Nano-ZS, 633 nm He-Ne laser) with 173^o^ detection angle. Attenuated total reflection - Fourier transform infrared (ATR-FTIR) spectra were measured by Nicolet iS50 FTIR from ThermoFisher. X-ray diffraction (XRD) patterns were obtained using Bruker D8 Discover high resolution X-ray diffractometer with Cu Ka radiation source (wavelength λ = 1.54 Å). Wide-Angle X-Ray Scattering (WAXS) was performed in Advanced Light Source Beamline 7.3.3, X-ray energy = 10 keV.

**Synthesis of polymer**

In a typical procedure for synthesizing PFO, a 20 mL flask was charged with 2,7-Dibromo-9,9 (di(oxy-2,5,8-trioxadecane))fluorene (2.0 g, 3.15 mmol), triphenylphosphine (82 mg, 0.315 mmol), 99.998% zinc powder-100 mesh (0.65 g, 9.5 mmol), 2,2-dipyridyl (24.5 mg, 0.165 mmol), and nickel chloride (8 mg, 0.065 mmol) under an Argon atmosphere. Subsequently, 2 mL of Dry DMAc was injected using a syringe, and the resulting mixture was stirred at 80 °C for a duration of 3 days. Following cooling to room temperature, the reaction slurry was diluted with 5 mL of additional tetrahydrofuran (THF). This mixture was then introduced into an 80 mL mixture of methanol and 50% HCl solution (25/75). The resultant polymer pellets, which formed as a precipitate, were collected through centrifugation at 6000 rpm. These pellets were then subjected to a purification process involving repetitive dissolution in THF and subsequent precipitation in water, which was repeated three times. The purified polymer pellets were further treated by undergoing three rounds of dissolution in THF and precipitation in hexanes. The molecular weight of produced PFO was analyzed by gel permeation chromatography (GPC). Mn = 11.0 kDa, PDI = 2.6.


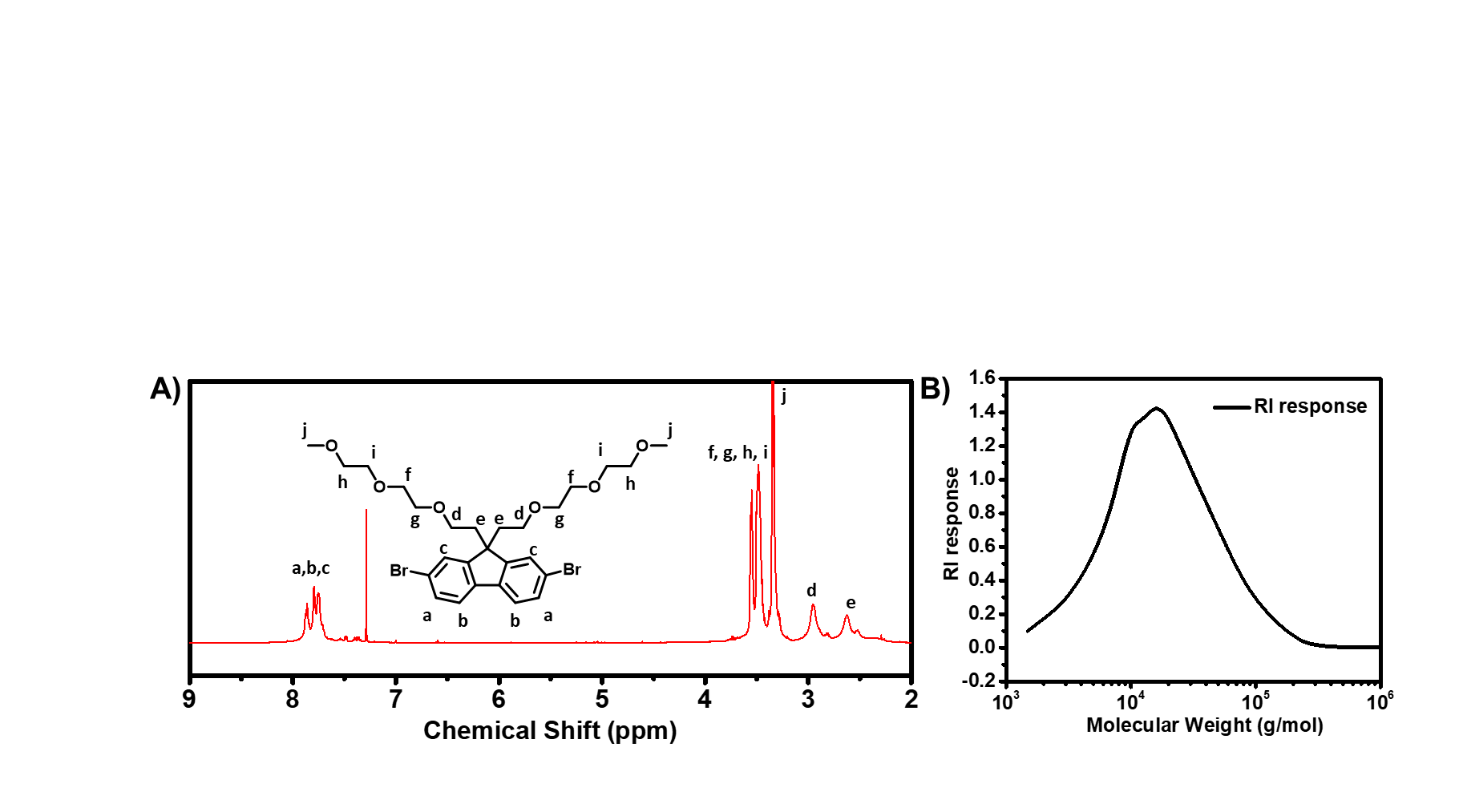


**Figure S1.** A) ^1^H-NMR spectrum of PFO in CDCl_3_. B) The molecular weight distribution of PFO.


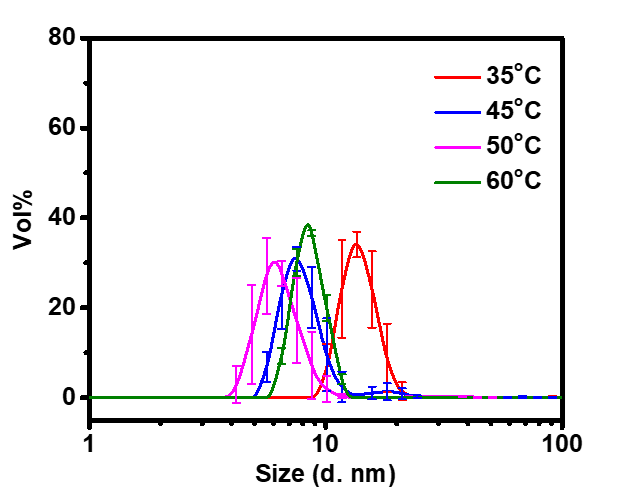


**Figure S2.** Hydrodynamic size evolution of PFO dispersion in EtOH/H_2_O mixture upon increasing temperature. We believe the solubility of PFO with oligo(ethylene glycol) side chains is also influenced by its lower critical solution temperature (LCST). Minimal aggregation was observed as the temperature approached their LCST.


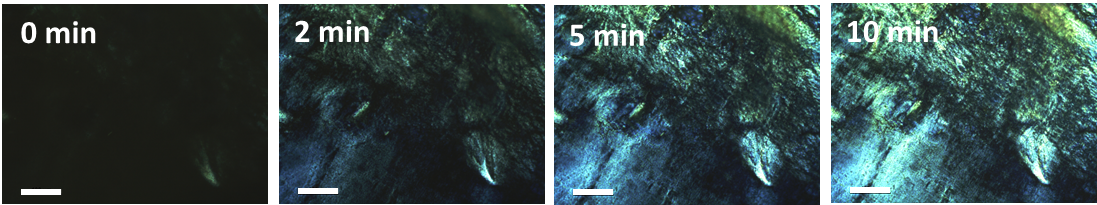


**Figure S3.** The evolution of polarized light microscopy (PLM) images of 10wt% PFO in ethanol/water 2:1 mixture upon air-drying. Scale bar: 100 µm.


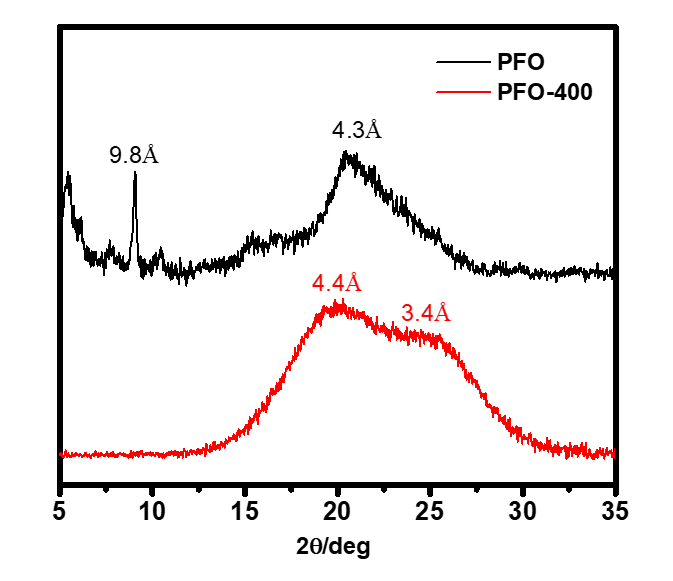


**Figure S4.** XRD patterns of PFO samples processed from chlorobenzene, as well as the produced PFO-400 after thermal processing at 400^o^C.


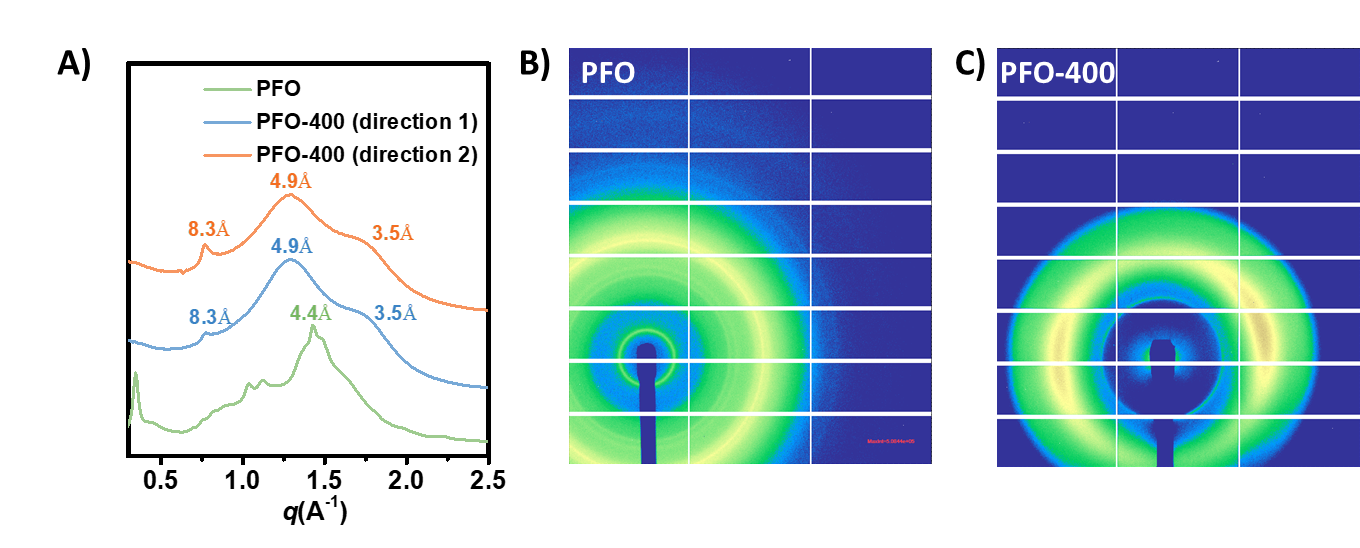


**Figure S5.** A) 1D Wide-Angle X-ray Scattering (WAXS) profile and B, C) 2D WAXS patterns of PFO samples processed from chlorobenzene, as well as the produced HOS-PFO after thermal processing at 400^o^C.


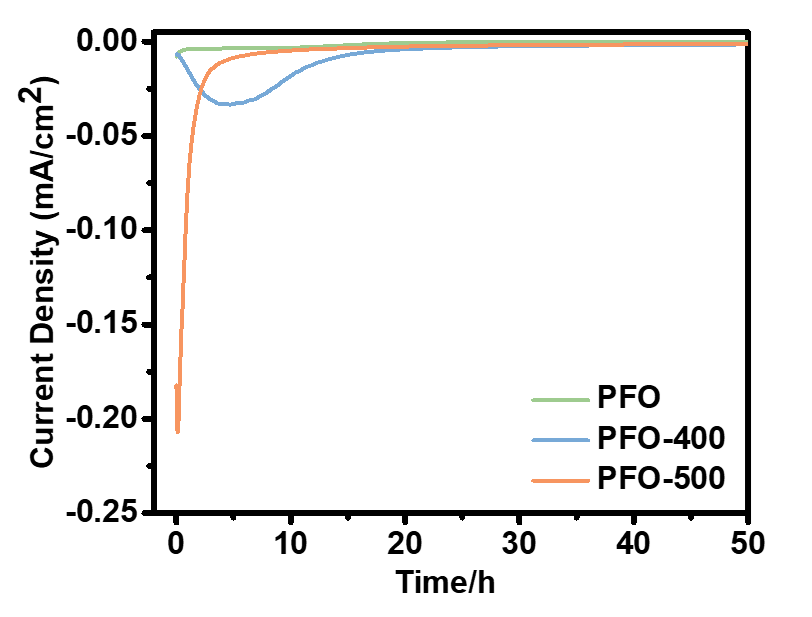


**Figure S6.** The current decay of PFO and HOS-PFO samples during potential hold experiment. 0.5 mg PFO ethanol/water solution was drop-casted on stainless steel spacer, then dried in vacuum at room temperature (PFO), heated to 400^o^C (PFO-400) or heated to 500^o^C (PFO-500). Samples were assembled to coin cells (anode: lithium metal). The potential of the samples was initially adjusted to 0.01V through Linear Sweep Voltammetry (LSV) at 0.1 mV/s, then hold at 0.01 V.

**Doping and electronic conductivity measurement**

Initially, PFO was dissolved in hot ethanol/water to create a 10 wt% solution. Subsequently, 5 μL of this solution was deposited onto the interdigitated electrode and left to air-dry overnight. To eliminate any residual solvent, the interdigitated electrodes underwent a vacuum oven drying process at 80°C for 12 hours. For electronic conductivity test of HOS-PFO, sample was prepared by further heating the PFO on interdigitated electrode, at 400^o^C or 500^o^C.

For testing the sample electronic conductivity under neutral state, sample was firstly soaked in Gen 2 electrolyte for 2 hours, then washed by EMC and dried by heat gun. The electronic conductivity was gauged through direct current circuit measurements of the interdigitated electrode. The voltage applied ranged from 20 mV to 100 mV. After that, lithiation of the sample was conducted in a two-electrode configuration. Lithium foil was positioned on the anode side, while the two pins of the interdigitated electrodes were initially short-circuited and then linked to the cathode side via copper wires. The potential of sample was reduced from open circuit voltage (OCV, ~3.0V) to 0 V through linear sweep voltammetry (LSV) with scan rate at 0.5 mV/s. The sample underwent further lithiation at 0 V versus Li/Li^+^ for different time period to achieve different doping status. The electronic conductivity at different doping status was measured using the same protocol described above.


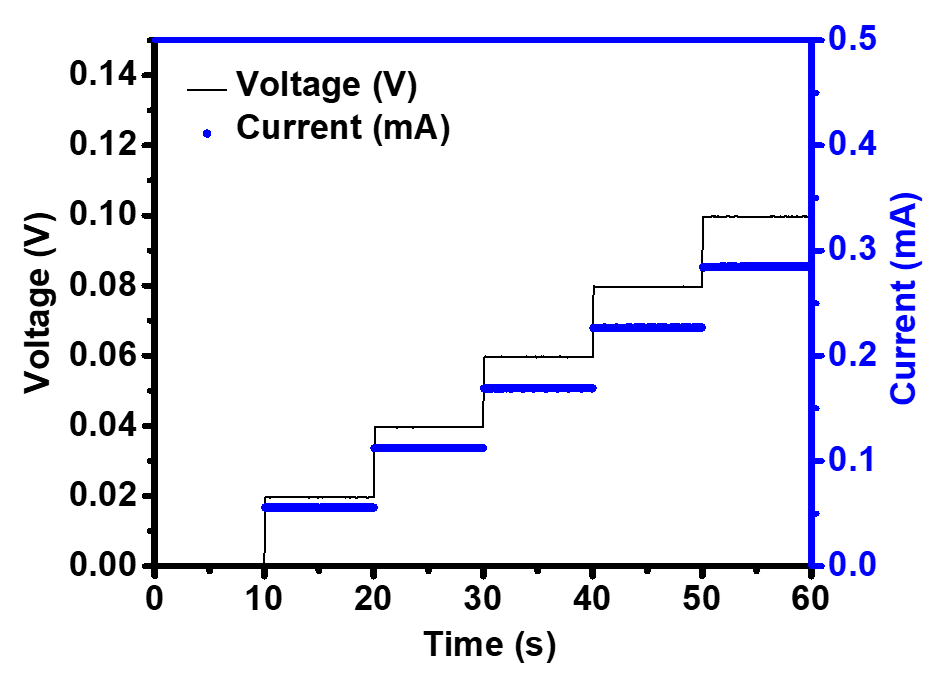


**Figure S7**. The DC test of PFO-400 (processed from water/ethanol) on interdigit electrode after final lithiation (OCV = 12 mV).

The electronic conductivity of HOS-PFO films at different lithiation status was calculated according to this equation^2^:


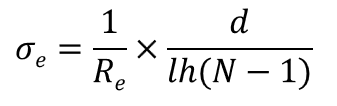


Re: Electronic resistance of the polymer; d: distance of interdigitated digits, 20 μm; N: numbers of interdigitated digits, 50; l: length of digits, 0.21 cm; h: thickness of gold coating (100 nm).

To evaluate the ionic conductivity of the HOS-PFO, the EIS measurement of the interdigitated electrode was performed with Lithium foil as the counter electrode. Rs (uncompensated solution resistance) and Zreal (real component of the impedance) were obtained from the Nyquist plot. Assuming rapid interfacial charge transfer and considering the ionic conductivity was much lower than the electronic conductivity, the electronic conductivity of HOS-PFO films at different lithiation status was calculated according to this equation:


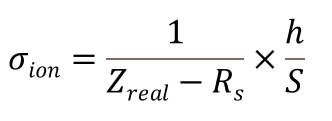


h: thickness of polymer coating (15 μm); S: area of the polymer coating.


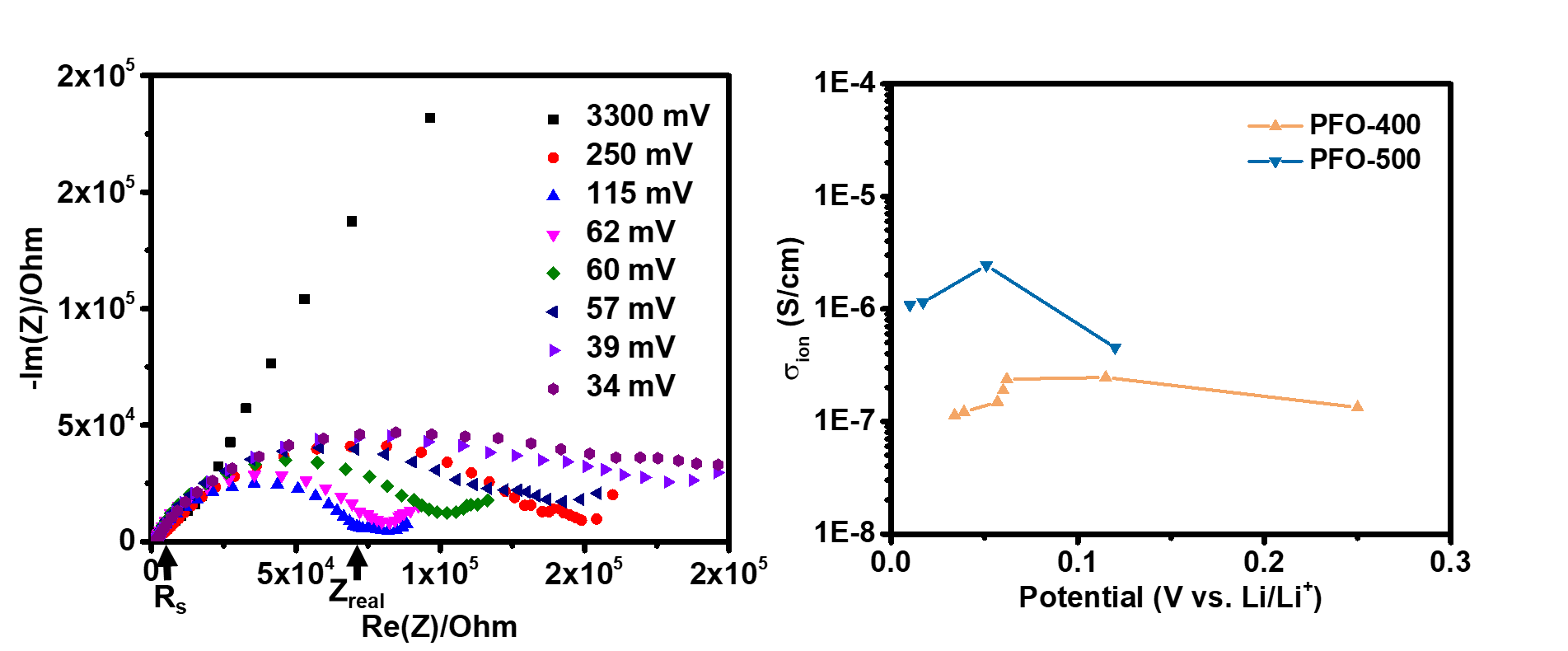


**Figure S8**. A) EIS of interdigitated electrode with loaded PFO-400 (processed from water/ethanol). B) The ionic conductivity of PFO-400 and PFO-500 at different potential.


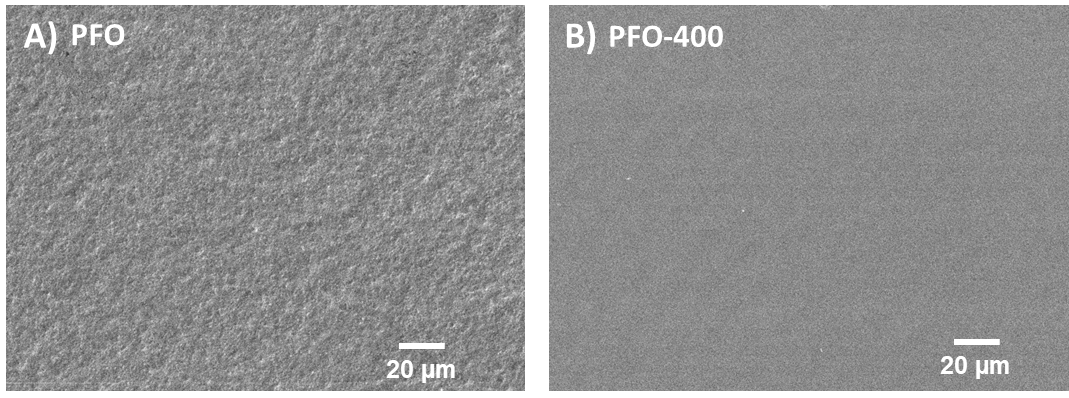


**Figure S9.** A) The surface morphology of PFO after being dried on Si wafer from NMP. B) The surface morphology of PFO-400 (processed from NMP).

**Fabrication of SiOx anodes with PFO/HOS-PFO as binder**

For half cell testing (1.0 mg SiOx/cm^2^)

Electrode preparation in chlorobenzene**:**

100 mg PFO was first dissolved in 1.5 mL of chlorobenzene under ultrasonication for 30 minutes. Then 200 mg SiOx composite was added to the solution and homogenized by continuous stirring. The obtained slurry was then coated on copper current collector with a common doctor blade coating method (wet gap = 100 μm) at room temperature. The produced SiOx-PFO electrode was dried at room temperature for 12 h and further dried by vacuum oven for 12 h, before being subjected to thermal processing for formation of hierarchically ordered structures. Typically, SiOx-PFO electrodes were placed in tube furnace under argon atmosphere protection. The temperature was ramped to different target temperatures (400^o^C or 500^o^C) in 2 hours, then held at the target temperature for 10 min. Electrodes (dia. = 9/16’’) were collected from the prepared films and stored in argon-filled glovebox before use.

Electrode preparation in ethanol-water mixture**:**

100 mg PFO was first suspended in 1 mL of 200 PRF ethanol under ultrasonication for 30 minutes. Then the suspension was heated to around 80^o^C which allows complete dissolution of the PFO polymers. Subsequently, 0.5 mL of pre-heated DI water (80^o^C) was added to the PFO solutions under stirring, followed by addition of 200 mg SiOx composite. The obtained slurry was then maintained at 80^o^C, before being coated on copper current collector with a common doctor blade coating method (wet gap = 100 μm). Surface was pre-heated to 50^o^C and the wet gap for doctor blade was controlled at 100μm or 200 μm. The produced film was dried at 50^o^C for 1 h and further dried by vacuum oven for 12 h. The thermal processing procedure employed here closely resembled that used for electrodes prepared from chlorobenzene.

For full cell testing (3.0 mg SiOx/cm^2^)

The preparation of the thicker electrodes designed for full-cell study is similar. The SiOx: PFO ratio in slurry was increased to 3:1 (300 mg SiOx and 100 mg PFO in 1.5 mL solvent), and we also increased the wet gap of doctor blade from 100 μm to 200 μm.


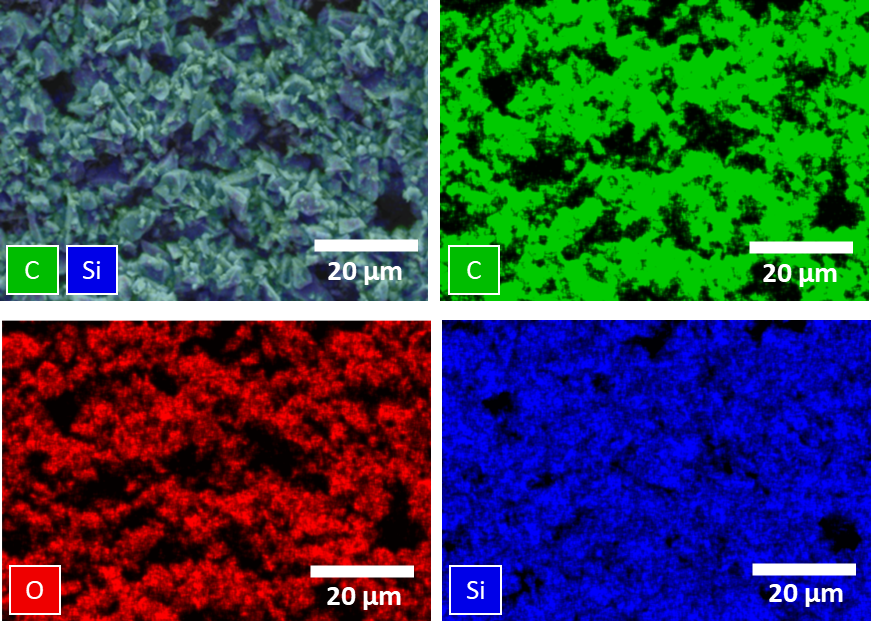


**Figure S10.** EDS-based elemental mapping result of SiOx-PFO-aq-500 (theoretical mass loading = 1.0 mg SiOx/cm^2^) fabricated from ethanol/water (v:v = 2:1)


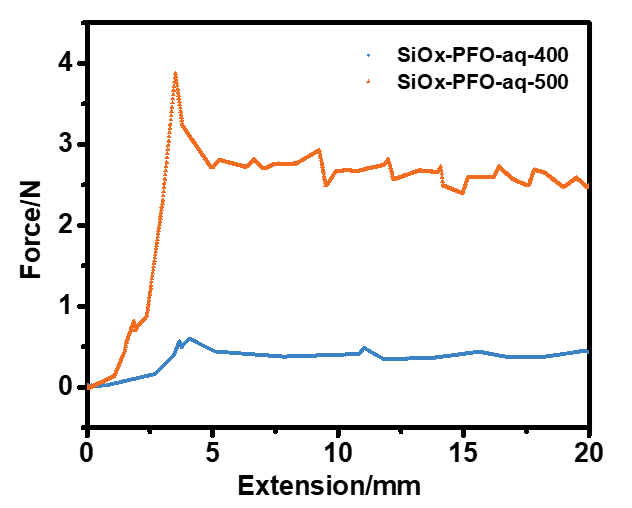


**Figure S11.** The 180^o^ peeling test results of the SiOx-PFO-aq-400 and SiOx-PFO-aq-500. (theoretical mass loading = 1.0 mg SiOx/cm^2^).


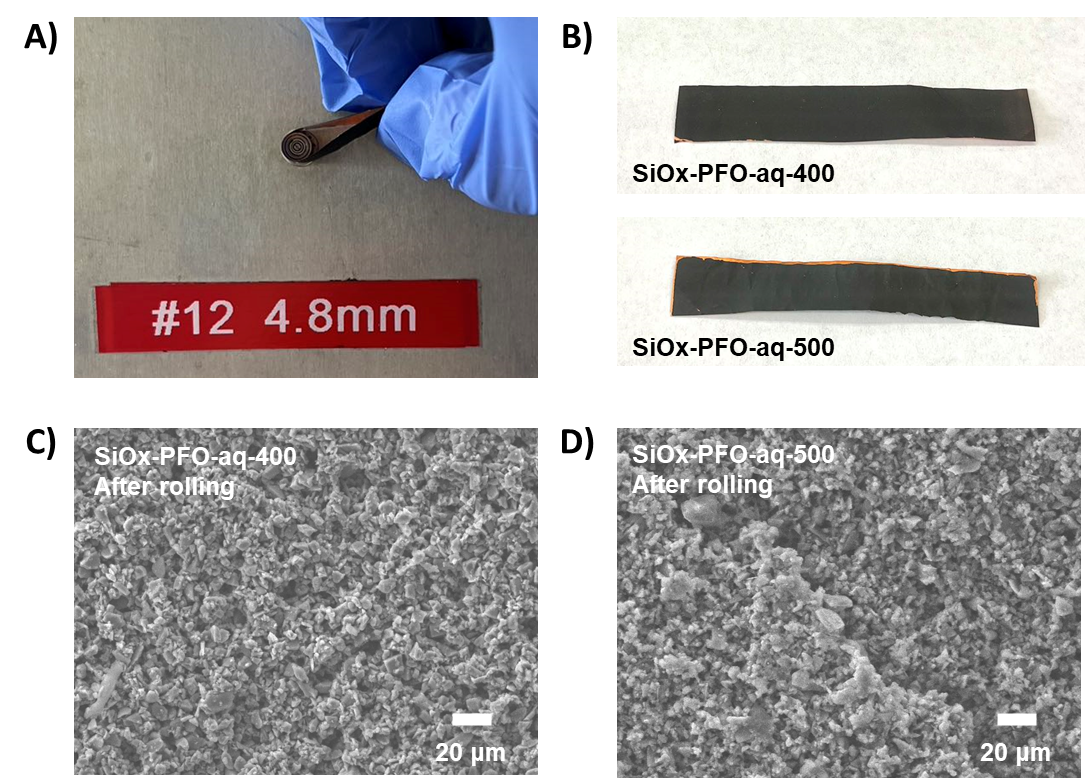


**Figure S12.** A) The roller setup for evaluation of electrode flexibility. B) The appearance of SiOx-PFO-aq-400 and SiOx-PFO-aq-500 (1.0 mg SiOx/cm^2^) after rolling testing for 20 cycles. C, D) The SEM images of SiOx-PFO-aq-400 and SiOx-PFO-aq-500 after rolling testing for 20 cycles.

**Cell assembly and battery cycling**

**Half Cells:**

CR2032 half-cells (Hohsen Co.) were assembled in Ar-filled glove box by sandwiching the separator (Celgard 2400) between prepared electrode (HOS-PFO weight% ranges from 19.8% to 16.4% depending on the thermal processing temperature) and lithium metal disk. Gen 2 electrolyte (1.2 M LiPF_6_ in ethylene carbonate: ethylmethyl carbonate (EC: EMC, 3:7 by weight)) was used as electrolyte. Around 40 μL electrolyte was used for individual cell. Cell formation was performed by cycling between 0.01V to 1.0V at C/10 rate for 3 times. After that, cell was cycled in between 0.05V to 1.0V.

**Full Cells:**

Pre-lithiation of SiOx-PFO anodes (~3.0 mg SiOx/cm^2^) was performed by half-cell electrochemical method. CR2032 half-cells were assembled in Ar-filled glove box by sandwiching the separator (Celgard 2400) between prepared electrode and lithium metal disk. Cell was cycled between 0.01V to 1.0V at C/20 rate and C/10 rate. In the last cycle, the cell was charged to 0.6 V to partially withhold lithium in the electrode (Figure S13). The cell was then disassembled in Argon-filled glovebox to obtain the pre-lithiated anode. Full cell was then assembled by sandwiching the separator (Celgard 2400) between pre-lithiated anode and NMC811 cathode (Areal capacity = 4.0 mAh/cm^2^). Gen 2 electrolyte was also used as electrolyte and around 40 μL electrolyte was used for individual cell. Full cell formation was performed by cycling between 3.0V to 4.1V at C/20 rate and C/10 rate. After that, the cell was cycled between 3.0V to 4.1V at C/3 rate.

*
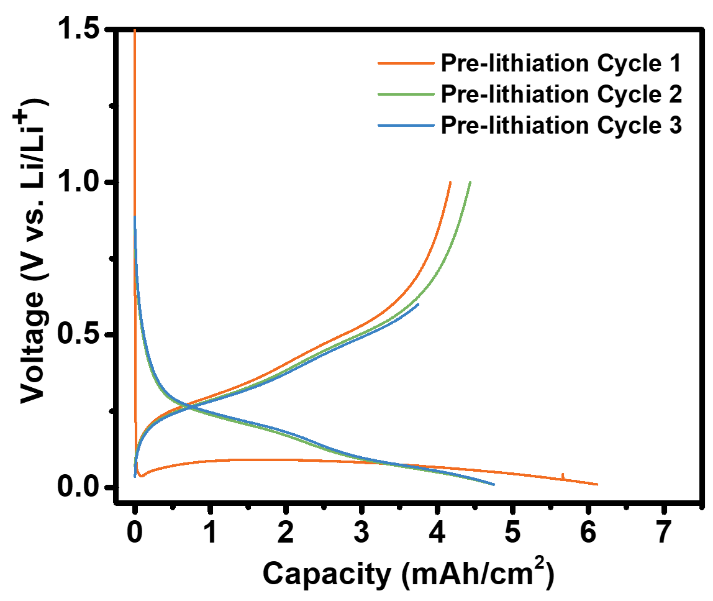
*

**Figure S13.** The charge-discharge profile of the electrochemical pre-lithiation process of SiOx-PFO-aq-400 anode (theoretical mass loading = 3.0 mg SiOx/cm^2^).


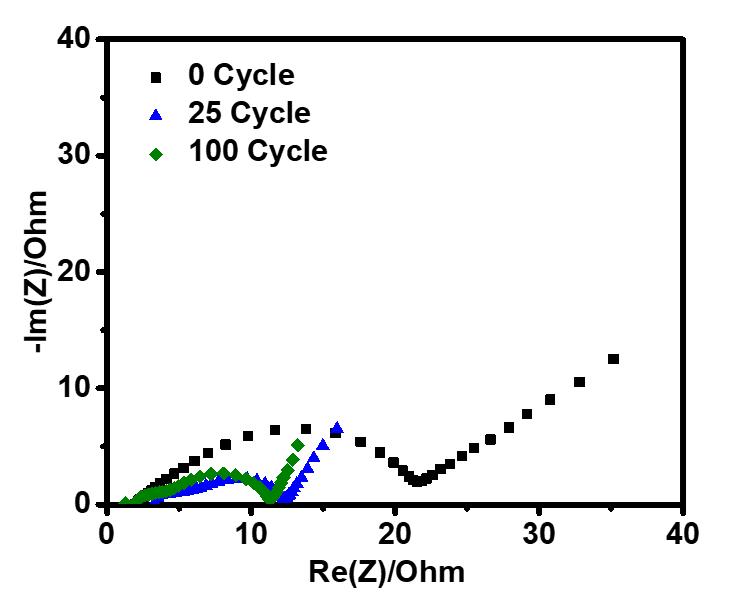


**Figure S14.** The EIS spectra of the Li||SiOx-PFO-aq-400 half cell during the cycling test.


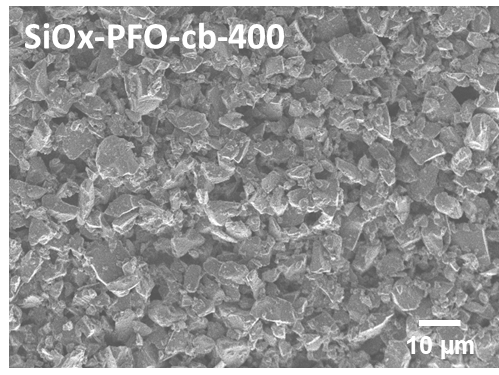


**Figure S15.** SEM image of the SiOx-PFO-cb-400 electrode (theoretical mass loading = 1.0 mg SiOx/cm^2^). Sample was prepared from chlorobenzene slurry coating and then heated to 400^o^C.


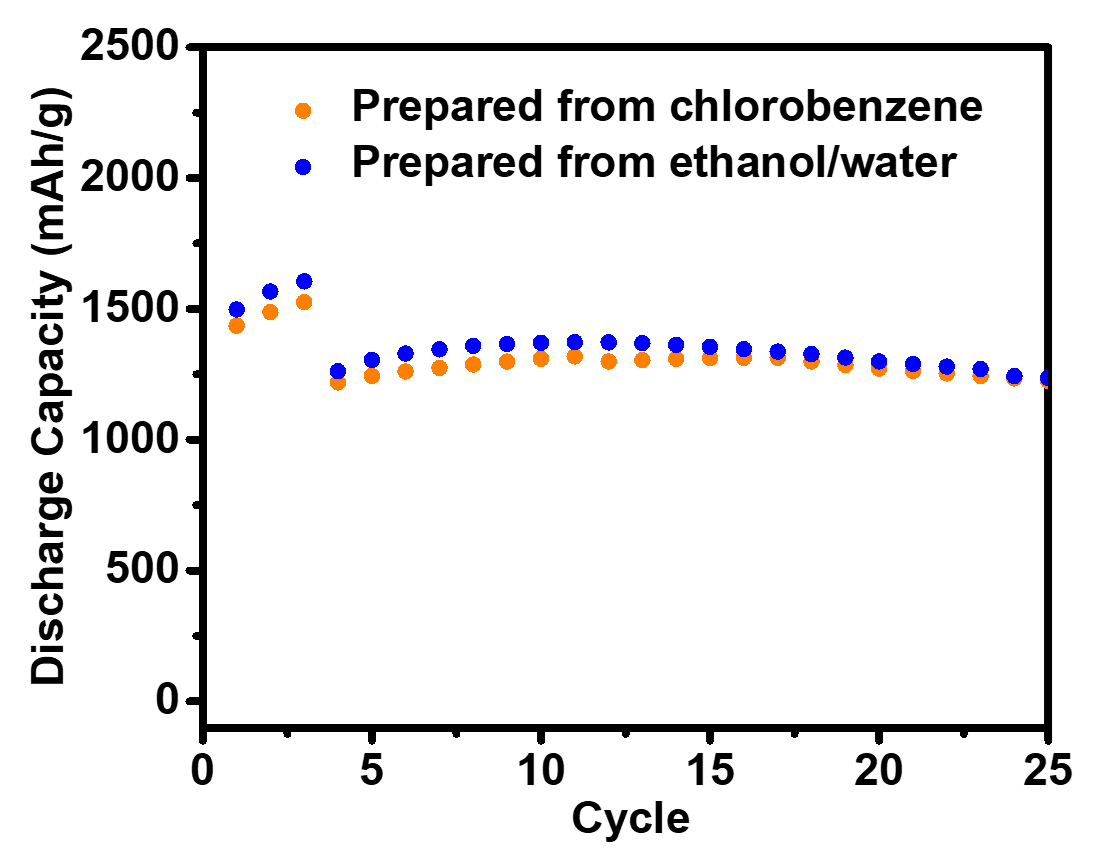


**Figure S16.** The capacity and Coulombic efficiency evolution of the Li||SiOx-PFO half cells (theoretical mass loading = 1.0 mg SiOx/cm^2^).


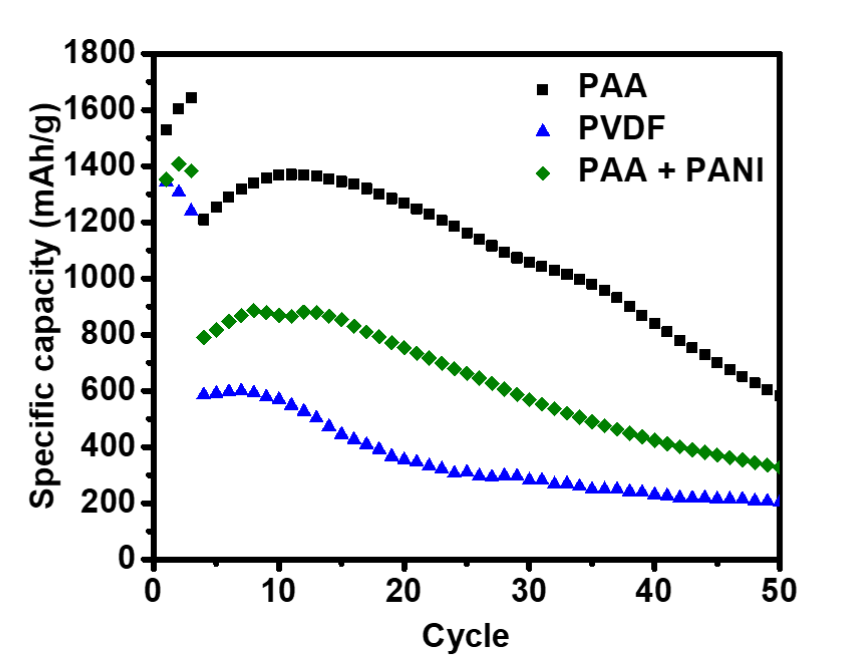


**Figure S17**. The evolution of specific capacity of SiOx-PAA, SiOx-PVDF and SiOx-PAA-PANI half cells. Formation of the cells was performed at C/10 rate for 3 cycles, followed by cycling test C/3 rate. Each electrode sample contained 60% SiOx, 20% graphite, 15% binder and 5% Super P carbon black.

**
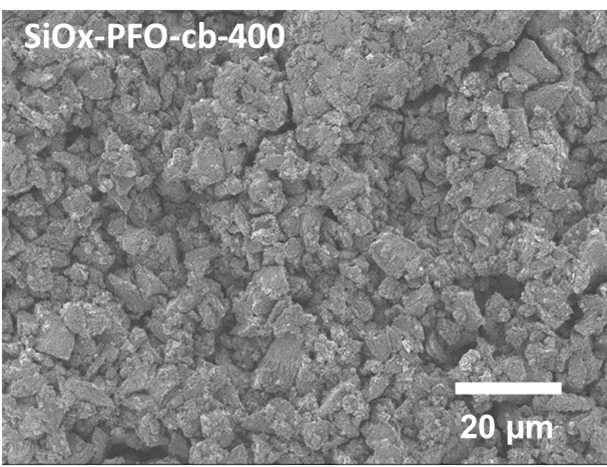
**

**Figure S18**. The surface morphology of SiOx-PFO-cb-400 samples (3.0 mg SiOx/cm^2^) after full cell cycling for 200 cycles.

**Table S1**. Comparison of the reported Si/SiOx anodes with conductive polymer binder

| Active material | Conductive polymer binder | Electrode Formulation | Electrolyte Formulation | Ref |
| --- | --- | --- | --- | --- |
| Si  (180 nm) | PEDOT: PSS | Si: Binder: Acetylene Black = 80:10:10 | 1.0 M LiPF_6_ in EC: DEC (1:1 by weight) with **5% FEC additive** | *Adv. Energy Mater.* **2018,** *8* (11). |
| SiOx  (microsized) | CCB | SiOx: Binder: Acetylene Black = 80:10:10 | 1.0 M LiPF_6_ in EC: DEC (1:1 by weight) with **10% FEC additive** | *Small* **2021,** *17* (42), e2102256. |
| Si (500 nm) | POD | Si: Binder: Carbon Black (Super C65) = 60: 20: 20 | 1.0 M LiPF_6_ in EC, DMC and DMSO  (5:5:3 v/v/v) **with 5 wt.% FEC additive** | *J. Mater. Chem. A*, **2021**, 9 (6), 3472-3481. |
| Si (<50 nm) | SSIP/FSIP | Si: Binder: Carbon nanotube=70:20:10 | 1.0 M LiPF_6_ in EC: DEC (1:1 by weight) **with 10% FEC and 1% VC additives** | *Adv Sci.* **2023**, 10 (6), e2205590. |
| Si (50 nm) | PANI | Si: Binder = 32: 68 | 1.0 M LiPF_6_ in EC: DEC (1:1 by weight) **with 5% FEC additive** | *Journal of Materiomics* **2023**, 9, 378-386 |
| SiOx (microsized, 5-10 μm) | HOS-PFO | SiOx: Binder = 85:15  **(No Conductive Additives)** | 1.2 M LiPF_6_ in EC: EMC (3:7 by weight)  **(No Additives)** | This Work |

**References**

1. Fung, B. M.; Khitrin, A. K.; Ermolaev, K., An improved broadband decoupling sequence for liquid crystals and solids. *J Magn. Reson.* **2000,** *142* (1), 97-101.

2. Zayat, B.; Das, P.; Thompson, B. C.; Narayan, S. R., In Situ Measurement of Ionic and Electronic Conductivities of Conductive Polymers as a Function of Electrochemical Doping in Battery Electrolytes. *J. Phys. Chem. C* **2021,** *125* (14), 7533-7541.
